# Metagenomic Characterization of the Human Intestinal Microbiota in Fecal Samples from STEC-Infected Patients

**DOI:** 10.3389/fcimb.2018.00025

**Published:** 2018-02-06

**Authors:** Federica Gigliucci, F. A. Bastiaan von Meijenfeldt, Arnold Knijn, Valeria Michelacci, Gaia Scavia, Fabio Minelli, Bas E. Dutilh, Hamideh M. Ahmad, Gerwin C. Raangs, Alex W. Friedrich, John W. A. Rossen, Stefano Morabito

**Affiliations:** ^1^Department of Food Safety, Nutrition and Veterinary Public Health, Istituto Superiore di Sanità, Viale Regina Elena, Rome, Italy; ^2^Department of Sciences, University Roma Tre, Rome, Italy; ^3^Theoretical Biology and Bioinformatics, Utrecht University, Utrecht, Netherlands; ^4^Centre for Molecular and Biomolecular Informatics, Radboud University Medical Centre, Nijmegen, Netherlands; ^5^Department of Medical Microbiology, University of Groningen, University Medical Center Groningen, Groningen, Netherlands

**Keywords:** STEC, microbiota, human gut, HUS, diarrhea, metagenomics

## Abstract

The human intestinal microbiota is a homeostatic ecosystem with a remarkable impact on human health and the disruption of this equilibrium leads to an increased susceptibility to infection by numerous pathogens. In this study, we used shotgun metagenomic sequencing and two different bioinformatic approaches, based on mapping of the reads onto databases and on the reconstruction of putative draft genomes, to investigate possible changes in the composition of the intestinal microbiota in samples from patients with Shiga Toxin-producing *E. coli* (STEC) infection compared to healthy and healed controls, collected during an outbreak caused by a STEC O26:H11 infection. Both the bioinformatic procedures used, produced similar result with a good resolution of the taxonomic profiles of the specimens. The stool samples collected from the STEC infected patients showed a lower abundance of the members of *Bifidobacteriales* and *Clostridiales* orders in comparison to controls where those microorganisms predominated. These differences seemed to correlate with the STEC infection although a flexion in the relative abundance of the Bifidobacterium genus, part of the *Bifidobacteriales* order, was observed also in samples from Crohn's disease patients, displaying a STEC-unrelated dysbiosis. The metagenomics also allowed to identify in the STEC positive samples, all the virulence traits present in the genomes of the STEC O26 that caused the outbreak as assessed through isolation of the epidemic strain and whole genome sequencing. The results shown represent a first evidence of the changes occurring in the intestinal microbiota of children in the course of STEC infection and indicate that metagenomics may be a promising tool for the culture-independent clinical diagnosis of the infection.

## Introduction

Shiga toxin-producing *Escherichia coli* (STEC) are a heterogeneous *E. coli* pathogroup causing food-borne outbreaks and sporadic cases of disease worldwide (Armstrong et al., 1996). STEC may cause severe afflictions in humans due to their ability to produce potent cytotoxins, the Shiga toxins (Stx), acquired upon infection with bacteriophages carrying stx genes, which can remain stably integrated into the bacterial chromosome (O'Brien et al., 1984). Stx exert their action by blocking the protein synthesis in the target cells by inactivating ribosomes (Okuda et al., 2006). Upon infection, the host can present a wide range of symptoms, including uncomplicated diarrhea, haemorrhagic colitis and the life-threatening haemolytic uremic syndrome (HUS).

The pathogenesis of STEC infections is not completely understood as it seems that, beside the virulence potential of the infecting strains, a number of other factors appear to be involved in the progression of the clinical symptoms. One possibility is that the human intestinal microbiota play a role by interfering with the ability of STEC to efficiently colonize the gastro-intestinal tract, as it has been proposed for other bacterial infections (Fujiwara et al., 2001; Gueimonde et al., 2007). Additionally, Gamage and colleagues proposed that different bacterial species in the host microbiota can act as amplifiers of the Stx-converting phage resulting in an augmented ability to produce the toxin (Gamage et al., 2003).

More and more data are becoming available on the role of human microbiota in health and disease and there is increasing evidence that the commensal bacteria play a crucial role in protecting human health (Forbes et al., 2016). Indeed, the human gut microbiota is a homeostatic ecosystem with several vital functions essential to host health, including protection against pathogens (Shreiner et al., 2015). Tap and colleagues showed that the human gut microbiota is governed by the presence of *Bacteroidetes, Firmicutes, Actinobacteria, Proteobacteria* and, in some cases *Verrucomicrobia* bacterial phyla (Tap et al., 2009). Members of these taxa, including *Faecalibacterium, Ruminococcus, Eubacterium, Dorea, Bacteroides, Alistipes*, and *Bifidobacterium* genera, constitute a phylogenetic core shared among individuals (Tap et al., 2009). In particular, it has been shown that species belonging to *Bifidobacterium* genus and butyrate-producing bacteria, belonging to the *Clostridiales* order, might exert a variety of beneficial health effects (O'Callaghan and van Sinderen, 2016; Rivière et al., 2016). Hence, a decrease in the relative abundances of *Bifidobacterium* species in the human colon has been associated with several disorders, such as inflammatory bowel disease, Crohn's disease and ulcerative colitis, irritable bowel syndrome, colorectal cancer, and increased gut permeability (O'Callaghan and van Sinderen, 2016). The mentioned disorders lead to a general change in the gut microbiota composition, favoring also the colonization and proliferation of pathogenic microorganisms (de Vos and de Vos, 2012).

We used a shotgun metagenomic sequencing approach to investigate the taxonomic composition of the gut microbiota in fecal samples taken from patients suffering from STEC infection and compared the results with those found in samples from healthy controls. Additionally, we analyzed fecal samples from patients with Crohn's disease with and without evidence of infection with STEC and added the microbial profiles of these samples to the analysis in order to assess if any change observed in the composition of the gut microbiota in course of STEC infections may be related with a dysbiotic status rather than associated with the STEC infection itself.

## Materials and methods

### Samples origin

Fecal samples (*N* = 10) from children (ages 0–4) were collected during an outbreak of STEC infections in 2015 in a nursery in the province of Rome, Italy. During the investigation, a STEC O26:H11 was isolated from different patients. Three samples were from patients with diarrhea (A.9, A.8, A.14), three were collected from patients after 2 weeks from the restoration of the normal intestinal function (A.40, A.32, A.41), and four samples (A.4, A.30, A.16, 481-5) from healthy subjects. In addition, four stool samples from four patients (ages 10–18) suffering from Crohn's Disease (CD) and hospitalized at the University Medical Center Groningen, The Netherlands, were included in the analysis (Samples 1, 2, 5, and 6). These samples were previously analyzed by real-time PCR for the presence of virulence genes associated with STEC (*stx1* and *stx2*) and EPEC (*escV*) (Gauthier et al., 2003) pathogroups, revealing the presence of *stx2* gene in Sample 2, the presence of the *escV* gene in Sample 1 and 5, and none of the mentioned virulence genes in Sample 6. The CD samples used for the present analyses were collected in the course of routine diagnostics and infection prevention controls. Oral consent for the use of such clinical samples for research purposes is routinely obtained upon patient admission to the UMCG, in accordance with the guidelines of the Medical Ethics Committee of the University Medical Center Groningen. The experiments, accordingly with the guidelines of the Declaration of Helsinki and the institutional regulations, were performed on anonymized samples.

### DNA extraction and sequencing

DNA of the specimens related with the Italian outbreak was extracted from 0.20 g of each stool sample using the EZNA Stool DNA extraction kit (Omega Bio-tek, Norcross, Ga.) following manufacturer's instructions, whereas for the CD samples, DNA was extracted from 0.25 g feces using the Power Soil DNA isolation kit (MO BIO Laboratories inc., Carlsbad, CA, USA) following the manufacturer's instructions. We did not observe marked differences in the purity of DNA produced with the two methods as assessed by considering the ratio between the absorbance measured at 260 and 280 nm.

Sequencing libraries were prepared from 100 ng of the DNA extracted from samples **A.9, A.4, and A.30**, using the NEBNext Fast DNA Fragmentation & Library Prep kit (New England BioLabs, New England, USA). In detail, the DNA was enzymatically fragmented to obtain fragments of about 400 bp, through an incubation at 25°C for 15 min, followed by 10 min at 70°C. The fragmented DNA was subjected to link with adapters and size selection of 450 bp fragments by electrophoresis on E-Gel SizeSelect 2% (Invitrogen, Carlsbad, USA) followed by PCR amplification as indicated in the NEBNext Fast DNA Fragmentation & Library Prep kit manual (New England BioLabs, USA). The libraries were amplified individually through emulsion PCR with an Ion OneTouch 2 and sequenced with an Ion Torrent Personal Genome Machine (Life Technologies, 118 Carlsbad, USA), using the 400 bp sequencing protocol. The three samples were sequenced individually in three different runs using a 316 V2 chip per run.

Sequencing of the samples **A.8, A.14, A.40, A.32, A.41, A.16, 481-5** as well as of the four CD fecal samples DNA was carried out using the TruSeq Nano DNA Library Preparation kit (Illumina, San Diego, CA, US) and a MiSeq platform (Illumina, San Diego, CA, US). In detail, sequencing libraries were prepared from 100 ng of the DNA extracted from each sample. DNA was mechanically fragmented to obtain a 350 bp insert size, using the M220 Focused-ultrasonicator™ based on Covaris AFA (Adaptive Focused Acoustics™) technology. The fragmented DNA was subjected to end repair and size selection of 350 bp fragments, followed by adenylation of 3' ends, link with adaptors and a final enrichment of DNA fragments, following the TruSeq Nano DNA Library Preparation kit manual (Illumina, San Diego, CA, US). The 11 samples were sequenced in three different runs, using a 600V3 cartridge per run generating 300 bp paired-end reads.

All the metagenomes are available at European Nucleotide Archive at EMBL-EBI under the accession number PRJEB23207.

### Bioinformatic analyses

#### Read mapping analysis

Reference-based bioinformatic analyses of the metagenomes were performed using the tools available on the ARIES public webserver (https://www.iss.it/site/aries/).

In detail, raw sequence reads were subjected to a quality check and trimmed to remove the adaptors and to accept 20 as the lowest Phred value. The identification of the presence of *E. coli* virulence genes was performed through the pipeline Virulotyper, which employs the Bowtie2 v2.3.2.2 program (http://bowtie-bio.sourceforge.net/bowtie2/index.shtml) (Langmead and Salzberg, 2012) to map the sequencing reads against the *E. coli* Virulence genes database (Joensen et al., 2014). Virulence genes showing average coverage above 1X were considered to be present in the sample.

Taxonomic analysis was performed using the DIAMOND v0.8.24 tool (Buchfink et al., 2015) to align the reads in FASTA format to the NCBI-nr (non-redundant) database (ftp://ftp.ncbi.nlm.nih.gov/) downloaded on July 2016, with the DIAMOND-BLASTX algorithm. Visualization was done with MEGAN (MEta Genome ANalyzer) version 6 (http://www-ab.informatik.uni-tuebingen.de/software/megan6/) (Huson et al., 2007).

The operational taxonomic units (OTUs) content of the samples was determined using MEGAN 6 and the converter script prot-gi2taxid-August2016X.bin downloaded from the NCBI website (https://www.ncbi.nlm.nih.gov). MEGAN 6 was also used to perform rarefaction analysis (Gotelli and Colwell, 2001).

#### Reference free analysis

The same metagenomes were also analyzed using a novel approach, as described by Kang et al. (2015), which allows assessing the degree of similarity between complex microbial communities through the reconstruction of draft genome sequences from shotgun metagenomic sequencing. Reads from the different metagenomes obtained with the Illumina platform were de novo assembled. The assembled genomic fragments were eventually grouped in putative genomes (bins) using probabilistic distances. For a visualization of the binning process we refer to Figure 1 in Kang et al. (2015). Default settings were used for all programs, unless otherwise mentioned.

In detail, the paired-end trimmed Illumina reads were cross-assembled with SPAdes v3.10.1 (http://bioinf.spbau.ru/spades) (Bankevich et al., 2012) in “—meta” mode (Nurk et al., 2017) and used as reference for mapping of all the single metagenomes, including those obtained with the Ion Torrent PGM. The quality of the cross-assembled scaffolds was evaluated using the QUality ASsessment Tool (QUAST) v4.5 (http://quast.sourceforge.net/) (Gurevich et al., 2013). QUAST provides assembly statistics including the number of assembled contigs, the length of the longest contig and the values of N50, N75, L50, L75, GC (%).

The reads of the metagenomes were mapped to the cross-assembled scaffolds using Burrows-Wheeler Aligner (BWA) v0.7.15, with the BWA-MEM algorithm (Li and Durbin, 2010). BWA-MEM was run with default settings, thus distributing reads that map to multiple places evenly, as suggested in the MetaBAT usage manual (https://bitbucket.org/berkeleylab/metabat). The output of BWA-MEM was converted from SAM format to the binary BAM format with the tools provided in the SAMtools suite (Li et al., 2009). The percentage of the reads mapping against the cross-assembly was estimated for the different metagenomes using SAMtools flagstat. The open source software MetaBAT2 (Metagenome Binning with Abundance and Tetra-nucleotide frequencies) v2.9.1 (http://bitbucket.org/berkeleylab/metabat) (Kang et al., 2015) was used to obtain bins from the scaffolds. MetaBAT2 was fed with the cross-assembly as input together with a depth file based on the bam files. The depth file was generated with the script jgi_summarize_bam_contig_depths, that is supplied with MetaBAT. The quality of the genomic bins generated was assessed with CheckM v1.0.5 (Parks et al., 2015) in “—lineage_wf” mode. CheckM assesses bin completeness, contamination, and strain heterogeneity based on the absence and presence of sets of expected single copy marker genes. Bins with a completeness >35% and a contamination <5% were selected for further analyses.

The scaffolds were annotated with the CAT (The Contig Annotation Tool) pipeline (Cambuy et al., 2016) and used to taxonomically classify the bins. To account for possible conflicts within a bin, we only annotated a bin to a taxonomic level if at least half of the length of the sequences within the bin showed a consistent annotation. If no annotation reached majority, we annotated the bin to the annotation with the longest length representation, but marked that annotation as “possible.” For instance, if 70% of the length of the bin is annotated on the genus level to *Escherichia*, but only 30% is annotated to *E. coli* on the species level, that bin is annotated as an *Escherichia* bacterium, possibly *E. coli*.

To calculate relative abundance of the bins in the samples, we used the depth file generated earlier with jgi_summarize_bam_contig_depths. Average coverage of a bin in a sample was calculated by multiplying the depth of the scaffolds in that bin by their length, and dividing their sum by the total base pair length of all the scaffolds in the bin. Abundance was made relative per sample by dividing this average read coverage per base pair by the sum of average read coverage per base pair for all the bins. Bin abundance was normalized to account for the fact that not all reads mapped to bins, by multiplying relative abundance with the fraction of binned reads, which was calculated as the sum of reads mapping to binned scaffolds (as calculated with samtools idxstats) divided by the total number of reads mapping to the cross-assembly (as calculated with samtools flagstat). Thus, the sum of relative abundances of all bins adds up to the fraction of total reads mapping to the cross-assembly that map to the bins in a sample.

The scaffolds were searched for the occurrence of the genes present in the *E. coli* Virulence genes database (Joensen et al., 2014). The database was queried with tblastx v2.6.0+ (Camacho et al., 2009) against the scaffolds. A gene was considered present on a scaffold if its hit had an e-value below 1e-5 and query coverage of at least 70%.

## Results

### Detection of virulence genes associated with pathogenic *E. coli* in the metagenomes

#### Detection of the STEC O26:H11 virulence genes

The virulence features of the STEC O26:H11 strain that had caused the outbreak had been previously identified by characterizing the isolated strain through real-time PCR and Whole Genome Sequencing (unpublished). In this work, we first checked the capability of metagenomics to identify the presence of the genes associated with the epidemic STEC strain. Mapping of the quality-controlled reads against the *E. coli* Virulence genes database (Joensen et al., 2014) showed the presence of all the genes composing the virulome of the STEC O26 outbreak strain (Table 1). In one sample (A.9), the metagenomics did not show the presence of the *espF* gene, while in sample A.14 we identified the presence of the gene *iroN*, which however was not present in the outbreak strain (Table 1). This gene was later identified in a scaffold that was annotated to the family *Enterobacteriaceae*, but was unbinned.

**Table 1:** Comparison of the STEC virulence genes identified in the samples collected from patients with STEC infection by metagenomics with those obtained through WGS of the isolated outbreak strain.

**Metagenomic analysis**										**Isolation based method**
**Patients with diarrhea**			**Recovered patients**			**Healthy subjects**				
**A. 9**	**A. 8**	**A. 14**	**A. 40**	**A. 41**	**A. 32**	**A. 4**	**A. 16**	**481-5**	**A. 30**	**Epidemic strain**
*Cif*	*cif*	*cif*	***ehxA***	*mchF*	***katP***	*epeA*	*cba*		*senB*	*cif*
*Eae*	*eae*	*eae*	***espP***	*tsh*	*lpfA*	***espP***	*cma*			*eae*
*efa1*	*efa1*	*efa1*	***katP***			*gad*	*gad*			*efa1*
***ehxA***	***ehxA***	***ehxA***	*mchC*			*iha*	*ireA*			***ehxA***
*espA*	*espA*	*espA*	*pic*				*iss*			*espA*
*espB*	*espB*	*espB*	*senB*				*lpfA*			*espB*
*espJ*	*espF*	*espF*	***toxB***				*pic*			*espF*
***espP***	*espJ*	*espJ*					*vat*			*espJ*
*gad*	***espP***	***espP***								***espP***
*iha*	*gad*	*gad*								*gad*
*iss*	*iha*	*iha*								*iha*
***katP***	*iss*	*iroN*								*iss*
*lpfA*	***katP***	*iss*								***katP***
*nleA*	*lpfA*	***katP***								*lpfA*
*nleB*	*nleA*	*lpfA*								*nleA*
*nleC*	*nleB*	*nleA*								*nleB*
*stx2a*	*nleC*	*nleB*								*nleC*
*tir*	*stx2a*	*nleC*								*stx2a*
***toxB***	*tir*	*stx2a*								*tir*
	***toxB***	*tir*								***toxB***
		***toxB***								

*Plasmidic genes normally present on the large virulence plasmid of STEC O26 are indicated in bold*.

The genes encoding the Stx were not identified in the specimens collected from healthy subjects and from recovered patients. However, we could observe in some of the latter metagenomes the presence of genes carried by the large virulence plasmid of STEC and identified also in the outbreak strain (Table 1). In particular, the metagenome from sample A.40 displayed the presence of plasmidic genes *ehxA* (Beutin et al., 1990), *espP* (Brunder et al., 1997), *katP* (Caprioli et al., 2005), and *toxB* (Tatsuno et al., 2000), while that from sample A.32 had the gene *katP* (Caprioli et al., 2005) (Table 1). Finally, we could not observe the presence of any gene associated with STEC in any of the metagenomes from healthy subjects, with the exception of the presence of *espP* in sample A.4 and *lpfA* in sample A.16 (Table 1).

The same analysis carried out on the metagenomes of the samples collected from patients with Crohn's disease confirmed the previous real-time PCR results (Table 2). In detail, Sample 6 appeared negative for the presence of virulence genes associated with pathogenic *E. coli* infections; Sample 2 showed the presence of the Stx2f encoding genes, confirming the previous evidence of STEC infection; finally, the remaining two samples, although they did not show the presence of the *escV* gene, previously identified by real-time PCR, displayed a virulence genes profile compatible with the presence of an aEPEC strain (Table 2).

**Table 2:** Comparison of virulence genes associated with pathogenic *E. coli* infection identified by metagenomics and real-time PCR in the samples collected from patients with Crohn's disease.

**Sample 1**		**Sample 2**			**Sample 5**		**Sample 6**	
**Metagenomics reads mapping**	**Real time PCR**	**Metagenomics reads mapping**	**Metagenomics bin alignment**	**Real time PCR**	**Metagenomics reads mapping**	**Real time PCR**	**Metagenomics reads mapping**	**Real time PCR**
*cba*	*escV*	*cif*	*eae*	*stx2f*	*cif*	*escV*		
*cif*		*cnf1*	*gad*		*espA*			
*cma*		*eae*	*hlyE*		*espF*			
*eae*		*espA*	*iha*		*gad*			
*espA*		*espC*	*ireA*		*nleB*			
*espJ*		*espF*	*iroN*					
*espD*		*espJ*	*katP*					
*gad*		*fim41a*	*lpfA*					
*iroN*		*gad*	*mchF*					
*iss*		*iha*	*pet*					
*katp*		*ireA*	*prfB*					
*IpfA*		*iroN*	*sat*					
*mchF*		*iss*						
*pet*		*lpfA*						
*tir*		*mchB*						
*tsh*		*mchC*						
*vat*		*mchF*						
		*mcmA*						
		*nleB*						
		*nleC*						
		*pic*						
		*stx2f*						
		*tir*						
		*vat*						

#### Detection of virulence genes in the cross-assembled scaffolds

In a complementary bioinformatic analysis of the same data, we assembled the metagenomic reads into scaffolds, and binned the scaffolds into draft genome sequences (Garza and Dutilh, 2015). The cross-assembly of the Illumina metagenomes, including the samples from STEC infections and the related control group as well as those from Crohn's disease, produced 429,862 scaffolds of size ≥500 bp, where the longest scaffold was 505,337 bp (Table S1). The percentage of the reads mapping against the assembled scaffolds was comparable between the different metagenomes, and seemed only slightly influenced by sequencing platform, ranging from 85.96 to 99.77% (Table S2). It is important to note that the cross-assembly is only based on the Illumina sequences, but the Ion Torrent reads showed high mappability values as well (Table S2). Finally, the metagenome binning produced 209 draft genome bins in total.

The tblastx search confirmed the presence of *E. coli* virulence genes in the cross-assembled metagenomic scaffolds, that were already detected in the metagenomes based on mapping the quality-controlled reads on the virulence genes database as described above. Additionally, this analysis allowed to localize each gene in specific scaffolds and within binned draft genomes. Most of the assembled scaffolds that contained virulence genes that were present in the epidemic strain were unbinned, but were annotated as *E. coli* on the scaffold level. Moreover, all of the plasmidic genes that were seen in recovered patients with read mapping were also unbinned. It is common that plasmids are not associated with draft genomes binned from metagenomes, as their genomic signals including abundance and nucleotide usage are different from those of the core genome (Beitel et al., 2014; Kang et al., 2015). Not all genes seen with read mapping were found in the scaffolds, the most notable omission being *ehxA*.

### Taxonomic profiling of the microbiota from stool samples

The metagenomic sequencing produced datasets of different sizes, mainly due to the use of two different sequencing platforms. As for the samples from STEC infections and the related control group, an average of 3,563,158 reads per sample were obtained from the Ion Torrent sequencing, while an average of 12,287,432 reads per sample were obtained from the Illumina platform. Nevertheless, the rarefaction curves (Gotelli and Colwell, 2001) showed that the diversity of taxonomic units was comparable between the different samples (Figure S1). The metagenomic sequencing of the four stool samples from Crohn's disease patients, produced with the Illumina sequencer produced on average 16,429,446 reads per sample.

#### Taxonomic profiling based on read mapping

**Figure 1:**
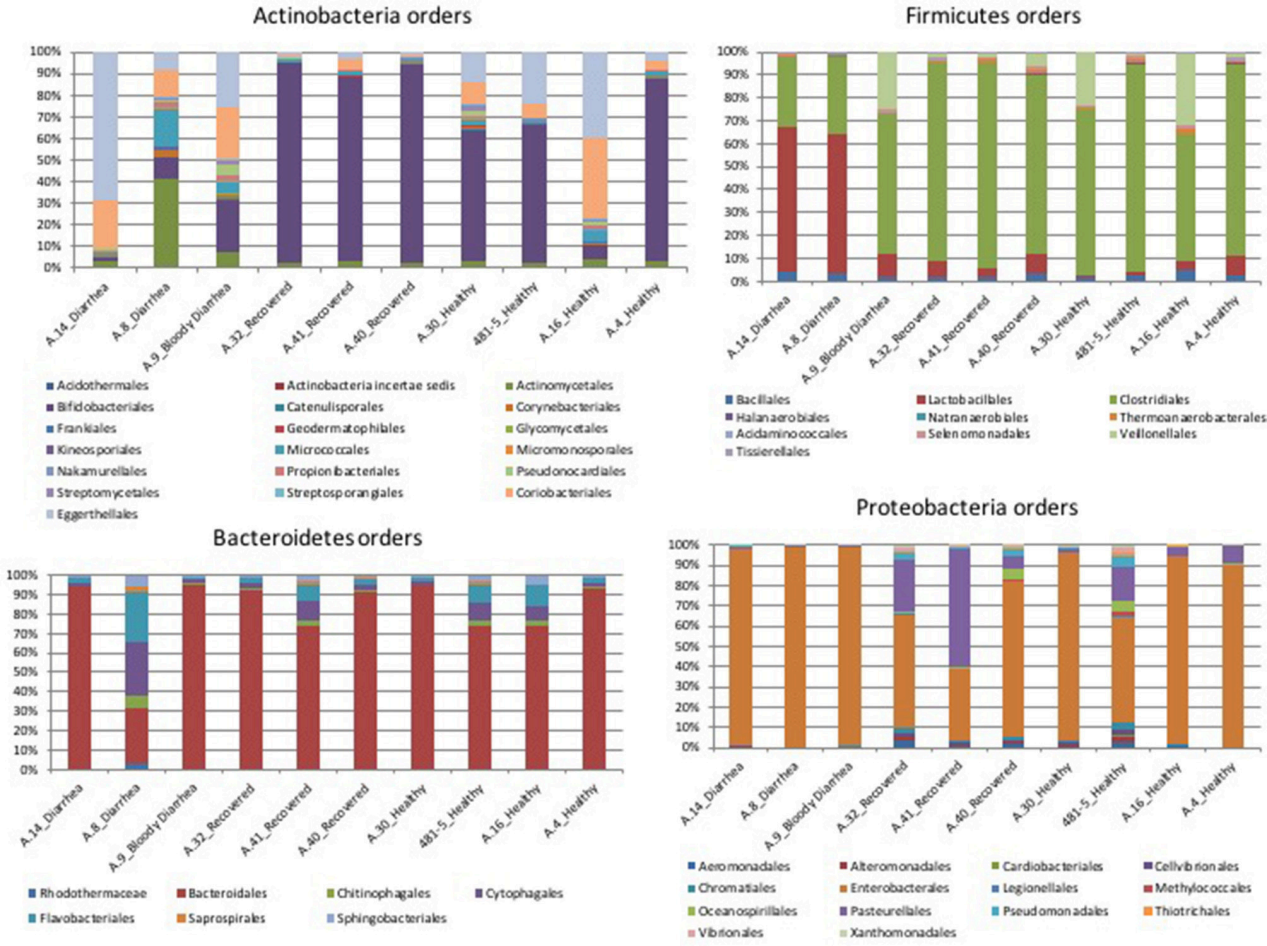
Distribution of the OTUs in the metagenomes from the STEC O26 outbreak, obtained through the analysis based on read mapping. The scale on the y axis refers to the percentage of the reads mapping to the specific OTU.

**Figure 2:**
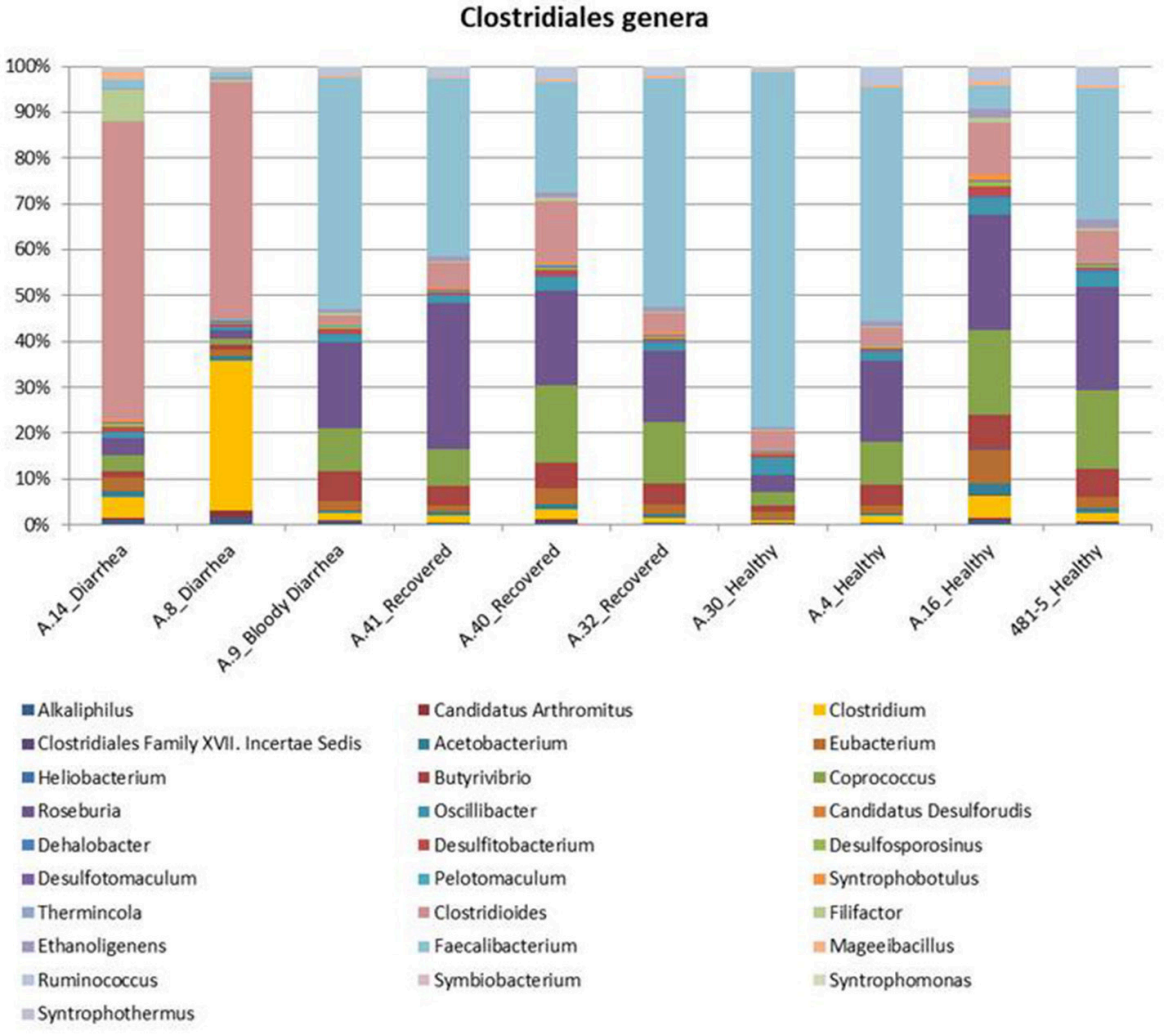
Distribution of the main genera belonging to the *Clostridiales* order in the metagenomes from the STEC O26 outbreak, obtained through the analysis based on read mapping. The scale on the y axis refers to the percentage of the reads mapping to the specific OTU.

In the metagenomes assayed, the most abundant phyla of *Bacteria* were the *Firmicutes* (16.6–79%), *Bacteroidetes* (0.2–63.1%), *Proteobacteria* (1.65–56.6%), and *Actinobacteria* (0.8–56.2%), whereas the phylum *Verrucomicrobia* showed a high abundance only in one sample (27.4% in A.30), obtained from a healthy subject (Table 3). In the samples analyzed the *Bifidobacteriales* order of the *Actinobacteria* phylum appeared more abundant in the vast majority of the STEC-negative samples (Figure 1) and a deeper analysis showed a marked prevalence of *Bifidobacterium longum* species (Figure S2). In addition to *Actinobacteria*, also some orders of the phylum *Firmicutes* showed a different distribution in the two groups of samples (Figure 1) (*p* < 0.05). The *Clostridiales* (*Clostridia*) were more abundant in the control group, while the *Lactobacillales* (*Bacilli*) predominated in the cases group (Figure 1) (*p* < 0.05) with the exception of sample A.9, which showed a taxonomic profile more similar to that of the control group with lower *Lactobacillales* (Figure 1). Moreover, the *Roseburia, Coprococcus, Butyrivibrio*, and *Faecalibacterium*, previously shown to be common in the intestinal microbiota from healthy subjects (Rivière et al., 2016; Hugon et al., 2017) were the most abundant genera of *Clostridiales* in the STEC negative samples assayed in this study (Figure 2), except for sample A.9, which again showed a profile more similar to those obtained from the control group samples. Members of the *Proteobacteria* and *Bacteroidetes* phyla apparently were not concerned by the perturbation of the intestinal microflora following the STEC infection as their relative proportions did not show patterns attributable to specific groups in the metagenomes analyzed (Figure 1).

**Table 3:** Relative abundance of the most abundant bacterial phyla in the samples from STEC-infected and healthy subjects, obtained through analysis based on read mapping.

	**A.9 (%)**	**A.8 (%)**	**A.14 (%)**	**A.40 (%)**	**A.41 (%)**	**A.32 (%)**	**A.4 (%)**	**A.16 (%)**	**481-5 (%)**	**A.30 (%)**
Actinobacteria	0.5	1.1	6.3	56.2	14.8	50	14	3.5	28.5	0.8
Proteobacteria	15.7	56.6	13.3	1.9	3.4	1.6	29.5	12.2	1.6	3.7
Bacteroidetes	63.1	0.2	7.46	6.6	1	11	3.2	5	1.3	51
Firmicutes	19.7	41.3	71.2	34.2	79	36.7	52	76.4	66.5	16.7
Verrucomicrobia	0.16	0.0	0.0	0.0	0.4	0.0	0.0	0.0	0.3	27.5

*Samples A.9, A.8, A.14 are from diseased patients; A.40, A.41, A.32 are from recovered patients; A.4, A.16, 481-5; A.30 are from healthy subjects*.

**Figure 3:**
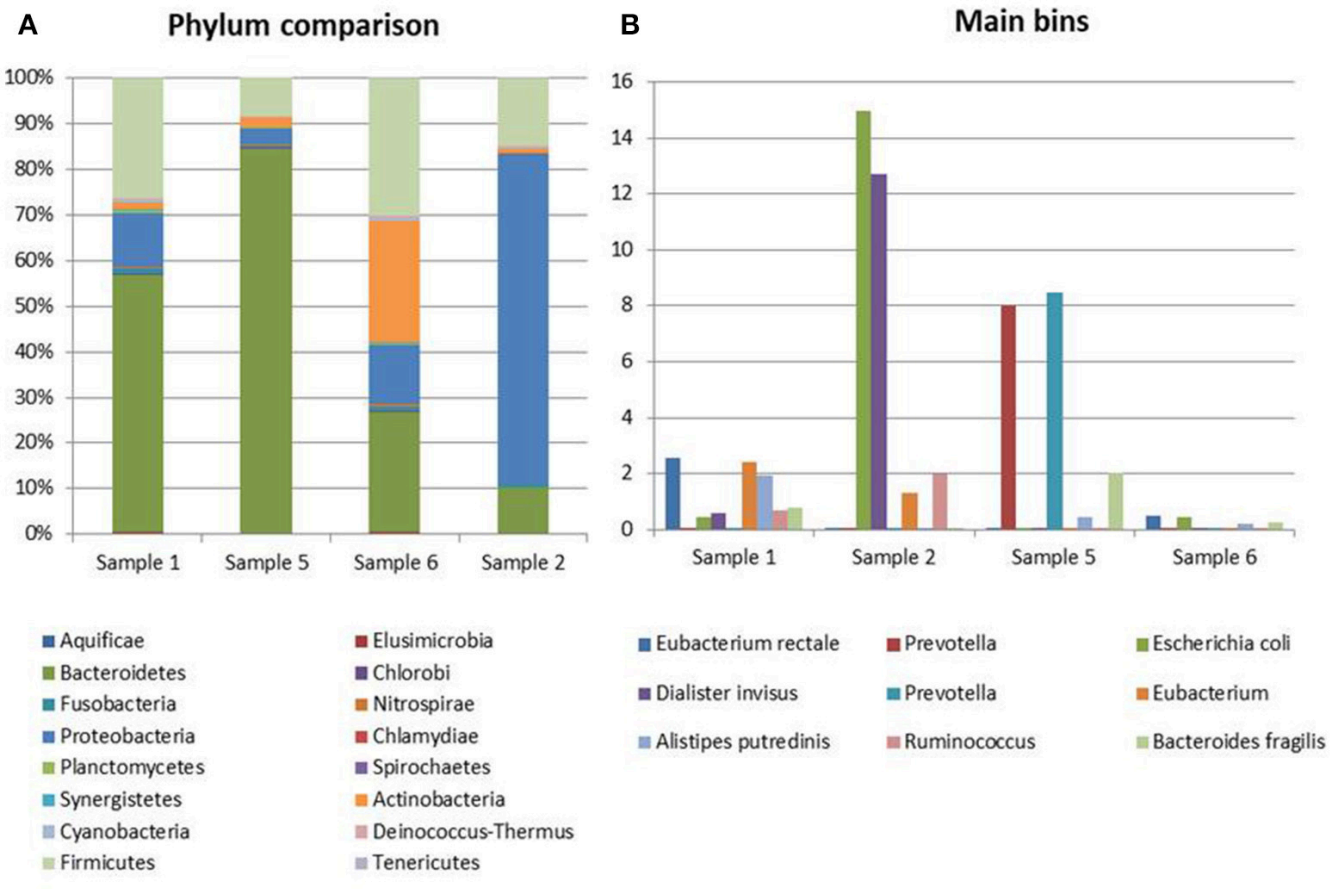
Taxonomic profiling of the samples from CD patients. **(A)** Distribution of the main bacterial phyla in the metagenomes obtained through the analysis based on read mapping. **(B)** Distribution of bins with an abundance value >2% (*N* = 10).

The most abundant bacterial taxa identified in the specimens collected from Crohn's disease patients were the *Bacteroidetes* (10.5–84%), *Proteobacteria* (4–77%), *Firmicutes* (9–29.5%), and *Actinobacteria* (1–26%) (Figure 3A). In one sample, a marked prevalence of the *E. coli* species was observed (Sample 2 in Figure 3). Interestingly, this sample was positive for STEC-associated genes, both in real-time PCR and at the metagenomic analysis.

#### Analysis of genome bins

For the analysis of the samples from STEC infections and the related controls, we selected 18 of the 209 bins based on values of completeness >35%, contamination <5%, and abundance >5% in at least one sample.

**Figure 4:**
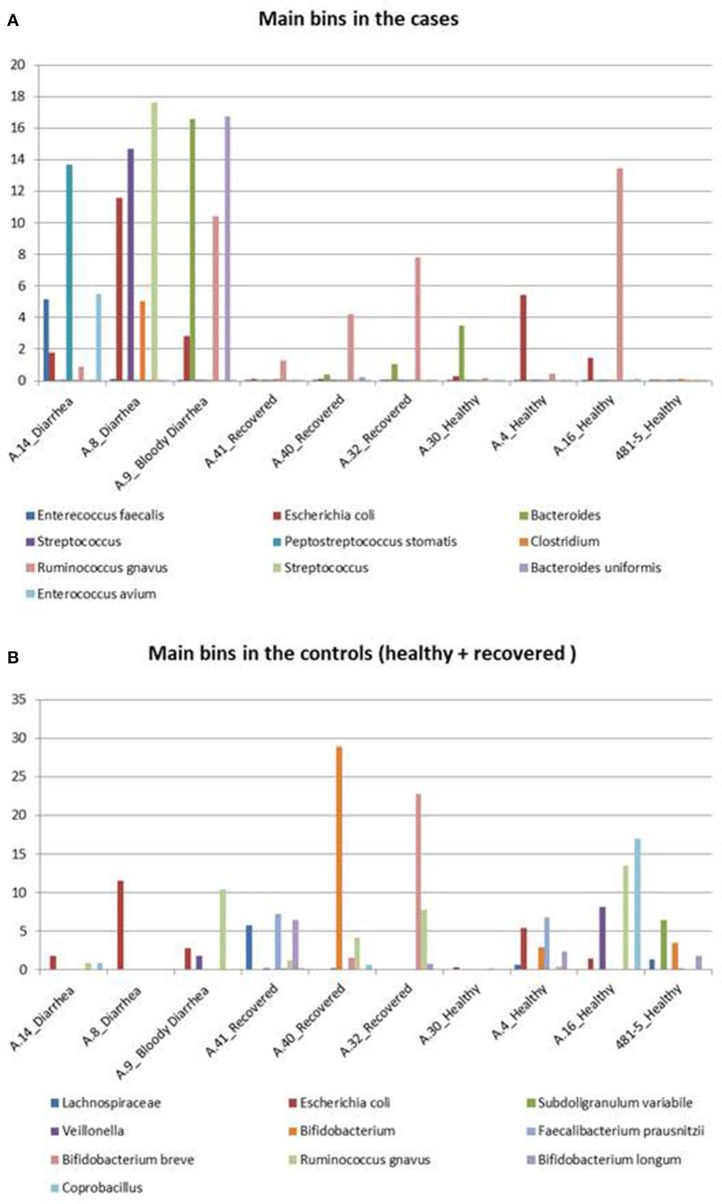
Taxonomic profiling of the metagenomes from the STEC O26 outbreak using a reference-free approach. The figure highlights the bins identified in the cases and controls. **(A)** Shows the distribution of the 10 bins more abundant in the cases group across all the samples, while **(B)** illustrates the 10 bins which are more represented in the controls group, including healed and healthy subjects across all the samples.

The abundance profile of the selected bins confirmed the results obtained with the reference-based approach, returning similar differences in the prevalence of specific taxonomic units between cases and controls (Figure 4).

In detail, *Enterococcus faecalis* and *Enterococcus avium* species and the *Streptococcus* genus, belonging to the order *Lactobacillales*, predominated in the STEC positive samples (Figure 4A). Concerning the *Clostridiales* order, members of *Peptostreptococcaceae* and *Clostridiaceae* families showed a high abundance in the cases group (Figure 4A), while *Lachnospiraceae* and *Ruminococcaceae* spp. (*Ruminococcus gnavus, Faecalibacterium prausnitzii*) showed a high representation in the set of samples from healthy and recovered subjects, confirming their association with a healthy status in the human intestine (Figure 4B).

Finally, the approach of reconstructing single genomes from complex microbial communities confirmed a clear prevalence of members of the *Bifidobacteriales* order in the control group (Figure 4B).

For the analysis of the metagenomes from Crohn's disease, we selected nine bins based on a completeness >35%, a contamination <5%, and an abundance value >2% in at least one sample.

This analysis confirmed the lower complexity of the intestinal microbiota in Crohn's disease and the high representation of *E. coli* species in Sample 2 identified with the read mapping approach (Figure 3B).

## Discussion and conclusion

The human gastrointestinal tract microflora, comprise ~10^14^ microbial cells that live in a mutual beneficial relationship with the host (Ley et al., 2006). Indeed, the gut microorganisms have a remarkable impact on human physiology, because they modulate the normal intestinal functions, produce vitamins and contribute to obtain energy from the food (Bäckhed et al., 2005). They have also a profound influence on the local and systemic immune responses and interfere with pathogen's colonization (Shreiner et al., 2015; Forbes et al., 2016).

In the present study, we aimed at using metagenomics as a tool to investigate possible changes in the composition of the intestinal microbiota in patients with STEC infection compared to healthy controls. Shotgun metagenomic sequencing was used to identify the presence of virulence genes associated with STEC strains and to perform a taxonomic classification of the microbial communities present in fecal samples collected from subjects involved in an outbreak caused by a STEC O26:H11 strain as well as from patients with Crohn's disease, with and without evidence of STEC and other pathogenic *E. coli* infections.

The identification of all the virulence features of the epidemic STEC strain in the metagenomes, previously determined through whole genome sequencing of the isolated outbreak strain (unpublished), showed that metagenomics is a promising diagnostic tool for infectious diseases, subjected to the availability of comprehensive databases of markers for the pathogens of interest. In sample A.14 the presence of the *iroN* gene, not present in the epidemic strain, was observed. The *iroN* gene encodes receptors involved in iron acquisition by bacteria (Russo et al., 2002), and it may have been present in a different co-circulating *E. coli* strain or even other bacteria.

The analyses carried out did not show the presence of genes encoding for Stx in specimens collected from both healed and healthy subjects, indicating a good specificity of the approach used. Interestingly, in samples A.32, A.40, and A.41 (healed patients) evidence of a previous STEC infection was provided by the presence of some genes know to be located on the large virulence plasmid of STEC (Table 1). It is possible that this plasmid may have remained in the bacterial population after the STEC strain was cleared, leaving traces of the previous STEC infection.

The analysis of the metagenomes obtained from patients with Crohn's disease also confirmed previously obtained real-time PCR results (Table 2). For Sample 1 and Sample 5 this approach failed to identify the *escV* gene, associated with Enteropathogenic *E. coli* (EPEC). However, in these samples the evidence of EPEC was provided by the identification of the presence of other genes characteristic of this pathogroup, but different from those used in the real-time PCR experiments (Table 2). Indeed, while the real-time PCR approach may be more sensitive, due to the exponential amplification of the targets, the metagenomic approach may complement the lower technical sensitivity with the simultaneous search for more determinants associated with the same pathogroup. The use of a wider panel of targets may confer a stronger ability to detect pathogens, but it requires the availability of accurate and curated databases with the genetic markers characteristic for any of the pathogen of interest.

The taxonomic profiling of the STEC-infected samples provided insight into the changes occurring in the intestinal microbiota upon STEC infection. As previously described by other authors (Tap et al., 2009), our results showed that the *Bacteroidetes, Firmicutes, Proteobacteria, Actinobacteria* were the bacterial phyla that prevailed in the samples, confirming their proposed role in maintaining the homeostasis in the human intestine (Tap et al., 2009) (Table 3). A deeper taxonomic classification highlighted a marked prevalence of some members of the *Bifidobacteriales* and *Clostridiales* orders in the STEC negative samples (Figures 1, 2, 4B). It has been already described that members of the *Bifidobacterium* genus confer positive health benefits to the human host (O'Callaghan and van Sinderen, 2016). These bacteria exert a probiotic action, stimulating the immune system (Perdigon et al., 1995), and providing a protection against gastrointestinal pathogens colonization by competitive exclusion of enteropathogens based on common binding sites on epithelial cells (Gueimonde et al., 2007). In addition, Fukuda and colleagues demonstrated that acetate production by *B. longum* strains is linked to the *in vitro* protection of host epithelial cells from the effect of Shiga toxin (Fukuda et al., 2011). The high abundance of *B. longum* and *B. breve* species, and *Bifidobacterium* genus observed in the samples collected from healthy and healed patients (Figure 4B) could have different explanations. Such a high proportion of *Bifidobacterium* genus in the intestine may have been effective in protecting the healthy subjects against the STEC infection or may have favored the positive outcome of the infection in the healed patients, which have not developed the HUS. On the other hand, the high abundance observed of the members of this genus could have been the effect of a cleared or absent infection.

Similarly, the high abundance of the *Clostridiales* order in the STEC negative samples and the opposite prevalence of *Lactobacilalles* order in the STEC positive samples could be put into relation with the STEC infection. As a matter of fact, it is possible that the intestinal colonization by STEC may contrast the normal permanence of *Clostridia* in the intestine, favoring the presence of *Bacilli*. It is interesting to note that *Faecalibacterium, Roseburia, Coprococcus*, and *Butyrivibrio* genera, highly prevalent in the metagenomes belonging to the control group, have all been proved to have a beneficial role in the human host intestine (Hugon et al., 2017). In this study, we have included some samples from patients with Crohn's disease. It has been described that patients with CD show a global decrease in the biodiversity of the fecal microbiota, essentially due to a markedly reduced diversity of *Firmicutes* (Manichanh et al., 2006; Sokol et al., 2008). As a matter of fact, a significantly reduced abundance of *Roseburia, Faecalibacterium*, and other genera belonging to the *Clostridiales* order in the intestine of CD patients has been reported (Kang et al., 2010; Morgan et al., 2012; Gevers et al., 2014). We considered the CD samples as showing taxonomic profiles related with a general status of dysbiosis not related with STEC infection and have evaluated the differences observed between the samples from the cases and the controls collected in the framework of the STEC outbreak in the light of the profiles observed in the dysbiotic CD specimens. Our results confirmed the low biodiversity of the gut microbiota in CD patients and the lower proportion of members of the *Firmicutes* phylum (Figure 3 and Figure S1). Additionally, our findings highlighted the absence of beneficial *Bifidobacterium* species in the feces collected from CD subjects, similarly to what was observed in the samples from STEC infections (Figures 1, 4). This latter observation, confirms that a decrease in the abundance of *Bifidobacterium* is associated with an infection with diarrheagenic agents or a dysbiosis status as it has been previously proposed (Gevers et al., 2014; O'Callaghan and van Sinderen, 2016; He et al., 2017), suggesting that the perturbation in the proportions of the *Bifidobacterium* genus observed in this study may not be specific for the infection with STEC.

Our results indicate that metagenomics is effective in detecting genomic traits associated with STEC in stool samples from infected subjects, making it a promising tool for the culture-independent diagnosis of the infections. Additionally, the bioinformatic procedures used can be automated and applied to the detection of other pathogens. The analysis of the taxonomic composition of the intestinal microbiota showed a good agreement between the data obtained with both the reference-free and read mapping approaches, supporting the following comparative analyses. In this respect, to the best of our knowledge, our data provide a first evidence of the changes occurring in the intestinal microbiota of children in the course of STEC infection. Further studies are required to assess the reasons underlying such differences and if certain taxonomic profiles may be considered effective in protecting the host from acquiring the STEC infection.

## Author contributions

FG performed the DNA extraction, the metagenomic sequencing, the bioinformatic analyses, and drafted the manuscript; FvM contributed to the bioinformatic analyses and revised the manuscript; AK installed the server for the bioinformatic analyses and provided assistance with the data analysis; VM contributed to the sequencing and bioinformatic analyses on the epidemic strain; GS contributed to the samples' seletion and critically revised the manuscript; FM contributed to the isolation of the epidemic strain; BD contributed to the bioinformatic analyses and revised the manuscript; HA collected the samples from Crohn' disease patients; GR contributed to the metagenomic sequencing; AF contributed to the metagenomic sequencing and revised the manuscript; JR contributed to the metagenomic sequencing and revised the manuscript; SM conceived the study and strongly contributed to revise the manuscript.

### Conflict of interest statement

The authors declare that the research was conducted in the absence of any commercial or financial relationships that could be construed as a potential conflict of interest.

## Abbreviations

STEC: Shiga toxin-producing *Escherichia coli*
HUS: Haemolytic Uremic Syndrome
CD: Crohn's Disease
EPEC: Enterophatogenic *Escherichia coli*
OTUs: Operational Taxonomic Units.
