# Supplementary material for: Metagenomic Characterization of the Human Intestinal Microbiota in Fecal Samples from STEC-Infected Patients
Source: Front Cell Infect Microbiol. 2018 Feb 6;8:25. doi: 10.3389/fcimb.2018.00025 (PMC5808120; doi:10.3389/fcimb.2018.00025)
Supplement: Supplementary file 1 [file Table1.DOCX]

**Table S1**. Assembly statistics indicating the quality of the cross-assembled scaffolds evaluated using QUAST.
