# Supplementary material for: Metagenomic Characterization of the Human Intestinal Microbiota in Fecal Samples from STEC-Infected Patients
Source: Front Cell Infect Microbiol. 2018 Feb 6;8:25. doi: 10.3389/fcimb.2018.00025 (PMC5808120; doi:10.3389/fcimb.2018.00025)
Supplement: Supplementary file 2 [file Table2.DOCX]

**Table S2**. Number and percentage of the reads from the 14 samples analysed in this study mapping against the cross-assembly. Samples A.9, A.4 and A.30 were sequenced with the Ion Torrent platform.

|  | **Number of the mapping reads** | **Percentage of the mapping reads** |
| --- | --- | --- |
| **A.9** | 3856842 | 90.93% |
| **A.8** | 11936333 | 98.95% |
| **A.14** | 12250589 | 98.82% |
| **A.32** | 14622057 | 99.59% |
| **A.40** | 11348934 | 99.60% |
| **A.41** | 13067306 | 99.35% |
| **481-5** | 8002827 | 99.20% |
| **A.4** | 2931083 | 87.13% |
| **A.30** | 3382512 | 85.96% |
| **A.16** | 16932979 | 99.71% |
| **Sample 1** | 20107751 | 99.63% |
| **Sample 2** | 15901940 | 99.46% |
| **Sample 5** | 19003584 | 99.77% |
| **Sample 6** | 12079692 | 99.23% |
