# Supplementary material for: Metagenomic Characterization of the Human Intestinal Microbiota in Fecal Samples from STEC-Infected Patients
Source: Front Cell Infect Microbiol. 2018 Feb 6;8:25. doi: 10.3389/fcimb.2018.00025 (PMC5808120; doi:10.3389/fcimb.2018.00025)

Figure S1. Rarefaction plot of the 14 metagenomes analysed in this study. The curves on the top of the chart correspond to the samples from the STEC O26 outbreak; while those present on the bottom part correspond to the samples from CD patients.

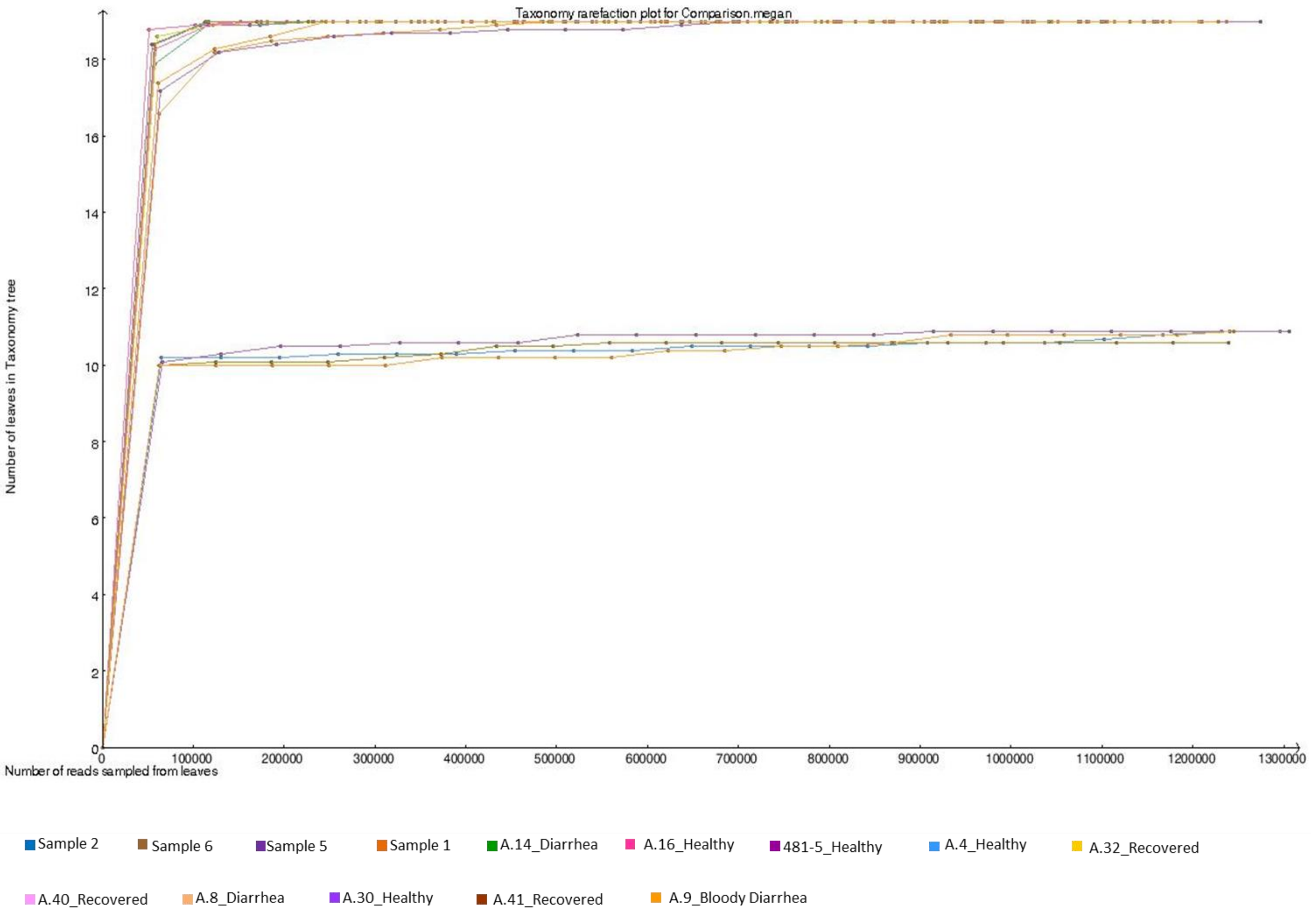

Supplement: Supplementary file 3 [file Image1.PDF]
