# Supplementary material for: Metagenomic Characterization of the Human Intestinal Microbiota in Fecal Samples from STEC-Infected Patients
Source: Front Cell Infect Microbiol. 2018 Feb 6;8:25. doi: 10.3389/fcimb.2018.00025 (PMC5808120; doi:10.3389/fcimb.2018.00025)
Supplement: Supplementary file 4 [file Image2.PDF]

Figure S2. Distribution of the species belonging to *Actinobacteria* phylum in the metagenomes from the STEC O26 outbreak, obtained through the analysis based on read mapping. The scale on the y axis refers to the percentage of the reads mapping to the specific OTU.

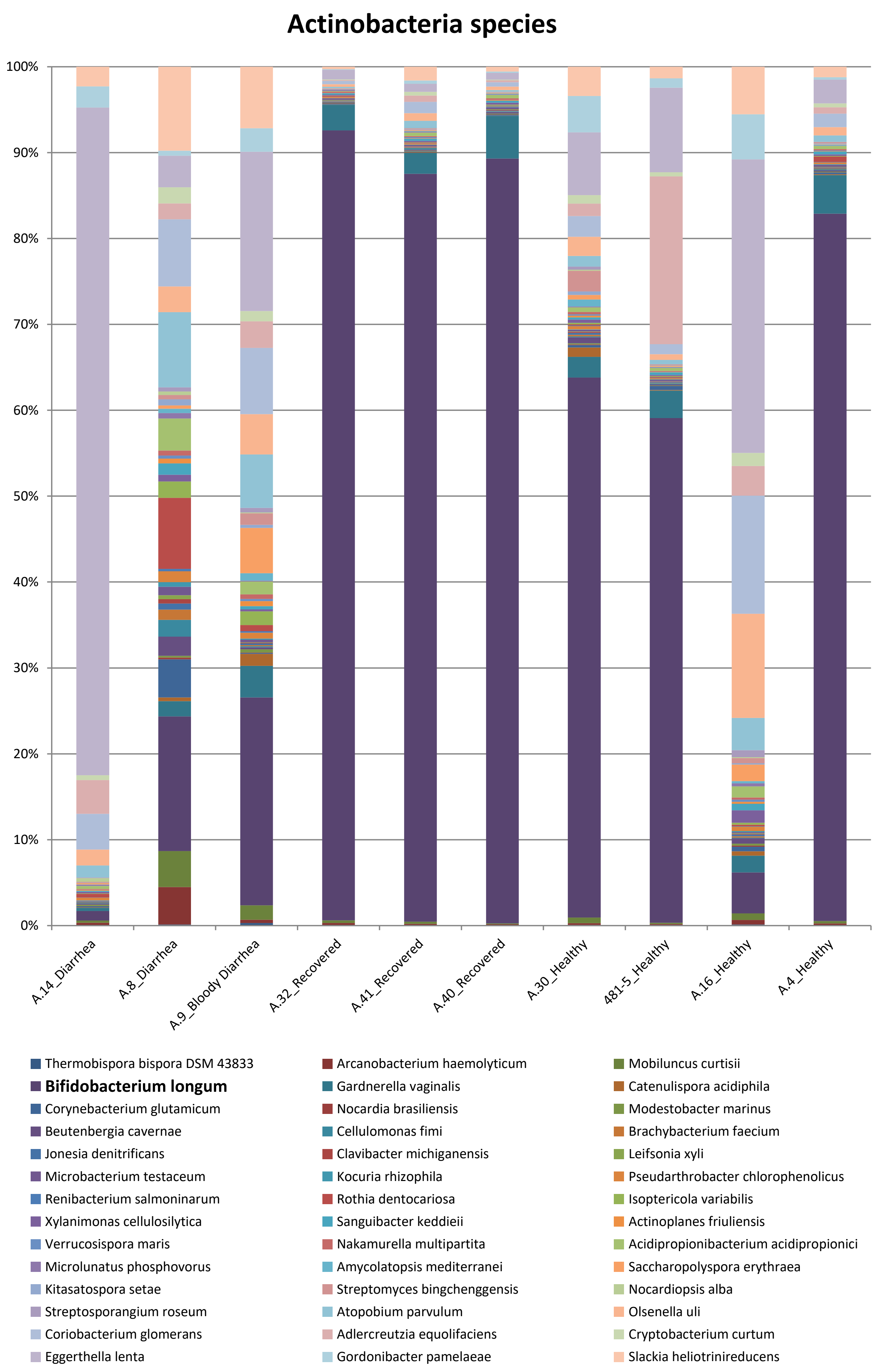
Actinobacteria species
100%
90%
80%
70%
60%
50%
40%
30%
20%
10%
0%
A.14_Diarrhea
A.8_Diarrhea
A.9_Bloody Diarrhea
A.32_Recovered
A.41_Recovered
A.40_Recovered
A.30_Healthy
481-5_Healthy
A.16_Healthy
A.4_Healthy
Thermobispora bispora DSM 43833
Bifidobacterium longum
Corynebacterium glutamicum
Beutenbergia cavernae
Jonesia denitrificans
Microbacterium testaceum
Renibacterium salmoninarum
Xylanimonas cellulosilytica
Verrucosispora maris
Microlunatus phosphovorus
Kitasatospora setae
Streptosporangium roseum
Coriobacterium glomerans
Eggerthella lenta
Arcanobacterium haemolyticum
Gardnerella vaginalis
Nocardia brasiliensis
Cellulomonas fimi
Clavibacter michiganensis
Kocuria rhizophila
Rothia dentocariosa
Sanguibacter keddieii
Nakamurella multipartita
Amycolatopsis mediterranei
Streptomyces bingchenggensis
Atopobium parvulum
Adlercreutzia equolifaciens
Gordonibacter pamelaeae
Mobiluncus curtisii
Catenulispora acidiphila
Modestobacter marinus
Brachybacterium faecium
Leifsonia xyli
Pseudarthrobacter chlorophenolicus
Isoptericola variabilis
Actinoplanes friuliensis
Acidipropionibacterium acidipropionici
Saccharopolyspora erythraea
Nocardiopsis alba
Olsenella uli
Cryptobacterium curtum
Slackia heliotrinireducens
